# A Possible Outbreak by *Serratia marcescens*: Genetic Relatedness between Clinical and Environmental Strains

**DOI:** 10.3390/ijerph18189814

**Published:** 2021-09-17

**Authors:** Giuseppina Caggiano, Francesco Triggiano, Giusy Diella, Francesca Apollonio, Marco Lopuzzo, Adriana Mosca, Stefania Stolfa, Carlo Pazzani, Marta Oliva, Carla Calia, Nicola Laforgia, Lidia Dalfino, Giovanna Barbuti, Pasquale Stefanizzi, Anna Maria Minicucci, Osvalda De Giglio, Maria Teresa Montagna

**Affiliations:** 1 Department of Biomedical Science and Human Oncology, University of Bari Aldo Moro, Piazza G. Cesare 11, 70124 Bari, Italy; francesco.triggiano@uniba.it (F.T.); giusy.diella@uniba.it (G.D.); francesca.apo@libero.it (F.A.); marcolopuzzo@gmail.com (M.L.); giovanna.barbuti@uniba.it (G.B.); pasquale.stefanizzi@uniba.it (P.S.); osvalda.degiglio@uniba.it (O.D.G.); mariateresa.montagna@uniba.it (M.T.M.); 2 Department of Interdisciplinary Medicine, Microbiology Section, University of Bari Aldo Moro, Piazza G. Cesare 11, 70124 Bari, Italy; adriana.mosca@uniba.it (A.M.); stolfastefania@gmail.com (S.S.); 3 Department of Biology, University of Bari Aldo Moro, Via Orabona 4, 70125 Bari, Italy; carlo.pazzani@uniba.it (C.P.); marta.oliva@uniba.it (M.O.); carla.calia@uniba.it (C.C.); 4 Neonatology and Intensive Care Neonatal Unit Section, Department of Interdisciplinary Medicine, University of Bari Aldo Moro, Piazza G. Cesare 11, 70124 Bari, Italy; nicola.laforgia@uniba.it; 5 Department of General Surgery, Gynecology and Anaesthesia—Anaesthesia and Intensive Care Unit II, University Hospital Policlinico of Bari, 70124 Bari, Italy; lidia.dalfino@yahoo.com; 6 Health Management, University Hospital Policlinico of Bari, 70124 Bari, Italy; annamariaminicucci@gmail.com

**Keywords:** *Serratia marcescens*, outbreak, intensive care unit, neonatal intensive care unit, genetic relatedness, pulsed-field gel electrophoresis, environmental sampling, strains

## Abstract

*Serratia marcescens* (SM) is a Gram-negative bacterium that is frequently found in the environment. Since 1913, when its pathogenicity was first demonstrated, the number of infections caused by SM has increased. There is ample evidence that SM causes nosocomial infections in immunocompromised or critically ill patients admitted to the intensive care units (ICUs), but also in newborns admitted to neonatal ICUs (NICUs). In this study, we evaluated the possible genetic correlation by PFGE between clinical and environmental SM strains from NICU and ICU and compared the genetic profile of clinical strains with strains isolated from patients admitted to other wards of the same hospital. We found distinct clonally related groups of SM strains circulating among different wards of a large university hospital. In particular, the clonal relationship between clinical and environmental strains in NICU and ICU 1 was highlighted. The identification of clonal relationships between clinical and environmental strains in the wards allowed identification of the epidemic and rapid implementation of adequate measures to stop the spread of SM.

## 1. Introduction

*Serratia marcescens* (SM) is a Gram-negative bacterium belonging to the family *Enterobacterales* that is commonly found in water, soil, animals, and plants [1,2]. Although SM was previously considered a saprophytic microorganism, its pathogenicity was demonstrated in 1913 [1,3] but, unfortunately, its role in the etiology of human infectious diseases was underestimated for many years [3]. The first outbreak of nosocomial infection caused by SM was confirmed in 1951, which then aroused the interest of many researchers [3]. Later, reports of infections caused by SM increased. Some authors [1,3,4,5] have shown that SM causes nosocomial infections in immunocompromised or critically ill patients admitted to intensive care units (ICUs). Other authors have reported that SM colonizes the respiratory and urinary tracts, especially in the presence of devices such as catheters and endotracheal tubes in adult patients [6]. Newborns, especially those admitted to neonatal ICUs (NICUs), also represent an important reservoir of SM. Once infected, many infants have persistent intestinal colonization that can last for several years, despite antibiotic treatment [1,7,8].

Because of its strong ability to adhere to invasive hospital equipment, SM can form biofilms and render conventional therapy ineffective [9], as a result of its intrinsic resistance to several classes of antibiotics (e.g., β-lactams and tetracyclines).

Data from the European Center for Disease Prevention and Control (ECDC) show that in 2017 *Serratia* spp. were ranked sixth among the ten most frequently isolated microorganisms in ICU-acquired pneumonia episodes (5.3%) and ninth in bloodstream and urinary tract infections (3.4%) in patients admitted to European ICUs [10].

The aims of this study were: (i) to evaluate the possible genetic correlation between clinical and environmental SM strains from the same hospital wards, and (ii) to compare the genetic profile of clinical strains with strains isolated from patients recovered in other wards of the same hospital.

## 2. Materials and Methods

From January to December 2020, 99 cases of SM infection occurred in a large Italian university hospital with 1400 beds in 33 separate buildings. The observational study involved patients admitted to internal medicine, endocrinology, infectious diseases, hematology, nephrology, general surgery, plastic surgery, cardiac surgery, and vascular surgery wards, NICU, ICU 1, and ICU 2; all located in different buildings. SM was isolated from 114 clinical samples, in particular: 46 (40.4%) from respiratory tract, 26 (22.8%) from urine, 15 (13.2%) from blood, 9 (7.9%) from effusion exudates, 6 (5.3%) from throat swabs, 4 (3.5%) from gastric aspirates, 3 (2.6%) from intravascular catheters, 2 (1.7%) from ocular conjunctiva, 2 (1.7%) from prostatic secretions, and 1 (0.9%) from cerebrospinal fluid. In most patients, SM was found in only one site but in 10 patients, SM was found in different sites.

In all wards, SM infections occurred sporadically except for those in the NICU and ICU 1. According to hospital protocols, in these cases (i.e., more than two episodes of illness caused by the same microorganism within a month in the same ward), it is mandatory to carry out environmental sampling to evaluate the possible presence of the same microorganism in the environment, especially on surfaces or equipment. Consequently, an environmental investigation was carried out in the NICU and ICU 1 to identify the possible environmental reservoir of infection.

### 2.1. Serratia marcescens in NICU and ICU

In September 2020, a newborn admitted to the NICU developed bacterial sepsis. Blood culture and central venous catheter (CVC) both tested positive for SM. The baby was preterm (25 weeks’ gestation) and, despite early antibiotic treatment, the newborn died 5 days after diagnosis of SM sepsis. During the same period in the same NICU, another preterm newborn developed SM sepsis that resolved after antibiotic therapy. At the same time, four patients developed SM infections in ICU 1. The microorganism was isolated from tracheal aspirates of three patients and from urine of the other patient.

The search for SM was performed by plating a loop of the biological sample on MacConckey Agar (Biolife Italiana srl, Milan, Italy) and incubating the plates at 37 ± 1 °C for 18–24 h. Identification of suspicious colonies was performed by matrix-assisted laser desorption/ionization time-of-flight mass spectrometry (MALDI-TOF MS, Biomèrieux, Marcy l’Etoile, France).

### 2.2. Environmental Sampling

One hundred and fifteen environmental samples were collected; 75 from the NICU (walls, incubators, changing tables, therapeutic trolleys, medical tools prewash brushes, washbasins, and jet breakers) and 40 from ICU 1 (walls, beds, bedside tables, therapy trolleys, sinks, and book supports). The sampling was performed by sterile swabs inserted in a plastic tube (Easy Surface Checking—Neutralizing Rinse Solution; Liofilchem Srl, Roseto degli Abruzzi, Italy) containing 10 mL transport medium, according to recommendations of the European Standard—International Organization for Standardization [11].

The flat and wide surfaces (regular surfaces, e.g., beds, bedside shelves, and incubators), were sampled with a swab on a well-defined area (10 × 10 cm), using a delimiter, while for small surfaces and curves (irregular surfaces, e.g., catheters, endotracheal tubes, and jet breakers) the available area was sampled. The swabs were placed in their protective cases, transported to the laboratory in refrigerated containers at 4 °C, and immediately tested. All samples were vortexed for 20 s at room temperature, and 1-mL aliquots of liquid transport medium of each dilution were mixed separately with Plate Count Agar (Microbiol Snc, Cagliari, Italy) to evaluate total microbial count, incubated at 30 ± 1 °C and checked daily for growth for 72 ± 3 h [12]. Colony counts were enumerated as colony-forming units (cfu) per cm^2^ (regular surface) or cfu/swab (irregular surface). At the same time, the search for SM was performed by plating a loop of the transport medium on Wurtz Agar (Biolife Italiana srl, Milan, Italy) and incubating the plates at 37 ± 1 °C for 24–48 h. Identification of suspicious colonies was performed by MALDI-TOF MS.

### 2.3. Antibiotic Susceptibility

The VITEK^®^2 automated identification system AST-N397 card (Biomèrieux, Marcy l’Etoile, France) was used to determine the antibiotic susceptibility patterns for amikacin, cefepime, ceftazidime/avibactam, ceftolozane/tazobactam, ciprofloxacin, colistin, gentamicin, imipenem, meropenem, tobramycin, and trimethoprim/sulfamethoxazole. Results were interpreted according to identification criteria set for *Serratia marcescens* by the European Committee on Antimicrobial Susceptibility Testing—EUCAST. The clinical and environmental strains were all sensitive to the antimicrobial drug classes tested.

### 2.4. Molecular Investigation

Molecular investigations by pulsed-field gel electrophoresis (PFGE) were carried out on clinical and environmental strains isolated in the NICU and ICU 1, and on other clinical isolates from patients admitted to different wards of the same hospital and available in our stock library. Four SM strains were isolated in ICU 2 (two from tracheal aspirates, and one each from bronchoalveolar lavage (BAL) and bronchial excretion); three in cardiac surgery (from blood cultures); and one in internal medicine (from blood cultures). Finally, two SM strains were isolated from BAL of patients admitted to general surgery.

### 2.5. PFGE

Genomic restrictions were performed as previously reported [13] and according to the standard operating procedure for PulseNet PFGE of *Escherichia coli* O157:H7, *E. coli* non-O157 (STEC), *Salmonella* serotypes, *Shigella sonnei*, and *Shigella flexneri* [14]. Agarose-embedded DNA was digested with 40 U *Xba*I for 4 h at 37 °C. The fragments were separated in 1% agarose gel (Pulsed Field Certified Agarose; BioRad, Milan, Italy) in Tris–borate–EDTA (44.5 mM Tris–borate, 1 mM EDTA; pH 8.0) at 14 °C, using a CHEF-DRIII (Bio-Rad, Milan, Italy) apparatus. Electrophoresis conditions were as follows: initial switch time 7.3 s, final switch time 24 s, voltage 6 V, included angle 120°, and run time 19 h. *Xba*I-digested DNA fragments from the *Salmonella* Braenderup strain H9812 were used in each gel as universal size standards. The agarose gels were stained with ethidium bromide (40 g/mL) and the DNA band images were acquired by the Gel Doc-It photo documentation system (UVP, Upland, CA, USA).

### 2.6. Genetic Relatedness

Genetically related isolates (clones) were indistinguishable from each other by genetic tests such as PFGE or were so similar that they were presumed to be derived from a common parent [15].

PFGE profiles were analyzed using the fingerprinting software GelJ [16]. Phylogenetic trees were constructed using the Dice coefficient with clustering by the unweighted pair-group method with arithmetic mean (UPGMA) and 2% tolerance in band position differences. The normalization of every gel image was performed using the *Xba*I-digested DNA fragments from *S*. Braenderup strain H9812 as an external reference. For determination of genotype diversity, a cutoff value of 80% was used [17] to interpret minimum similarity, while the strains were considered to belong to the same clonal group when PFGE profiles had a value of similarity ≥95%. The genetic correlation was first assessed on strains isolated from clinical and environmental samples in the NICU and ICU 1. These profiles were correlated with those of other clinical strains from patients in different wards of the same hospital.

The DNA restriction pattern that was designated the outbreak pattern was reported as type A; the isolates whose restriction patterns were indistinguishable from that pattern were reported as representing the outbreak strain. Patterns that were closely or possibly related to the outbreak pattern were considered as subtypes of A and were designated type A1, type A2, etc. Patterns that differed substantially from the outbreak pattern and were classified as unrelated were designated type B, type C, etc. [15].

## 3. Results

From environmental investigations carried out in the NICU and ICU 1, three swabs (4%, 3/75) in the NICU were positive for SM and two (5%, 2/40) in ICU 1. In the NICU, one strain was isolated from a brush used before sterilization of surgical forceps and two from washbasins, while in ICU 1, the two strains were isolated from a book support and a faucet handle.

Overall, 22 SM strains were subjected to molecular investigations: 17 clinical strains (three from the NICU, four from ICU 1, and 10 from other wards) and five environmental strains (three from the NICU and two from ICU 1) (Table 1).

### PFGE and Genetic Relatedness

Comparing SM clinical and environmental strains, based on the lower limit of similarity of 80%, 19 strains were grouped into three PFGE groups, designated A, B, and C, and in clonal subgroups A1, B1, B2, and C1 (Table 2).

Group A consisted of four strains, of which three (subgroup A1) were indistinguishable from each other: two clinical strains from NICU patient (blood culture and CVC) and an environmental strain isolated from a medical tools prewash brush from the same ward (100% similarity).

Group B consisted of nine strains: two in subgroup B1 and four in subgroup B2 (95% and ≥96% similarity, respectively). Subgroup B1 consisted of strains reported as cs-SM/10 and cs-SM/17 isolated from recovered patients in two different wards (general surgery and ICU 2). Subgroup B2 consisted of three strains indistinguishable from each other (100% similarity) and reported as cs-SM/14, es-SM/18, and es-SM/19. These strains came from ICU 1: two (es-SM/18 and es-SM/19) were detected from environmental samples (faucet handle and book support, respectively) and one (cs-SM/14) from tracheal aspirate. The fourth strain (cs-SM/20) was isolated from blood culture of a patient recovered in cardiac surgery.

Group C consisted of six strains. Two of environmental origin (es-SM/01 and es-SM/02, from two different washbasins in the NICU) constituted subgroup C1 (97% similarity). The other four strains, isolated from patients (blood culture and tracheal aspirate) admitted to different wards, did not belong to any PFGE subgroup.

**Figure 1:**
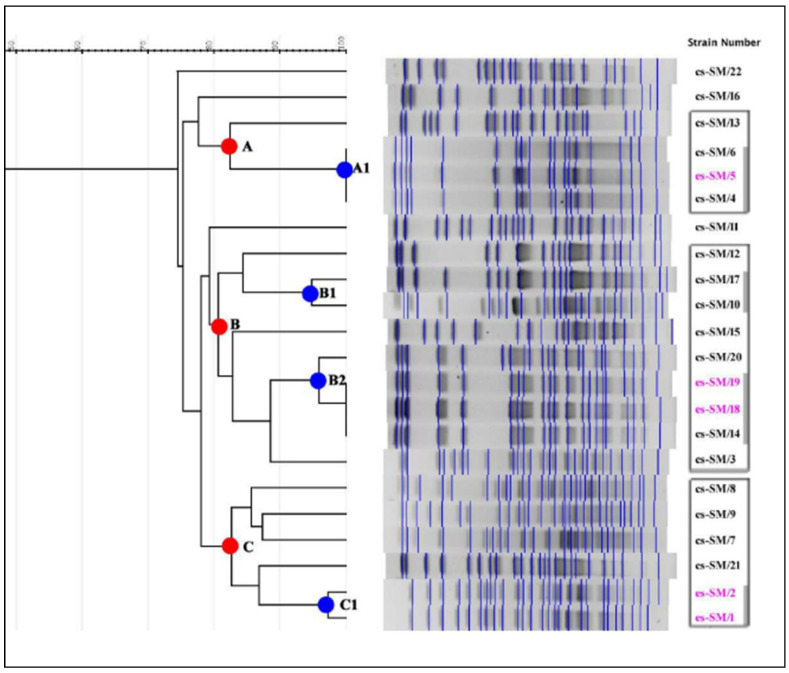
Pulsed-field gel electrophoresis generated dendrogram for 17 clinical and five environmental *Serratia marcescens* strains. Legend cs-SM (black color) = clinical strain-*S. marcescens*. es-SM (fuchsia color) = environmental strain-*S. marcescens*.

The last three strains (cs-SM/11, cs-SM/16 and cs-SM/22; all clinical strains) were labeled as unique PFGE profiles since their similarity to PFGE was ≤79%. In particular, cs-SM/11 and cs-SM/22 were isolated from tracheal aspirate and urine of patients recovered in ICU 1, and cs-SM/16 was isolated from BAL of a patient in general surgery (Figure 1).

## 4. Discussion

Although SM is an environmental saprophytic microorganism, it can cause dangerous nosocomial diseases in immunocompromised patients, as well as in wound and soft tissue infections [18], open burn surgical wound infections [19], and invasive burn wound infections [20].

Our study highlights distinct clonally related groups (A–C) of SM circulating between different wards and in different areas of the same hospital. The identification of three indistinguishable strains (subgroup A1) in the NICU, isolated from the CVC and blood culture of the same patient and from a medical tools prewash brush, emphasizes the possible cross-contamination between personnel and the environment, and how the latter may represent a potential source of microbial spread and a threat to human health. However, both clinical and environmental strains unrelated to subgroup A1 were also isolated in the same ward. A similar finding was observed in ICU 1 where indistinguishable strains (subgroup B2) from tracheal aspirates, faucet handles, and book supports and clinical strains unrelated to subgroup B2 were detected.

The presence of strains related to subgroups B1 and B2 was also highlighted in other wards. Strains cs-SM/10 and cs-SM/17, clonally related to subgroup B1, were detected in ICU 2 and general surgery wards, respectively, and cs-SM/20, clonally related to the B2 subgroup, was detected in cardiac surgery.

These data suggest the circulation in different wards of the same hospital of both clonally distinct and clonally related strains. The latter hypothesis could be consistent with the presence of environmental niches that act as reservoirs and sources of bacterial transmission. It would be interesting to investigate if this strain clonal relatedness might somehow be related to environmental and/or genetic features yet to be identified

The mode of transmission of SM from surfaces or patients is not easy to demonstrate. Cross-infection of SM, mainly from contaminated hands of hospital staff, has been highlighted for many years, as have environmental sources as risk factors for SM outbreaks [21,22,23]. The invasive disease may have been facilitated by the presence of CVC and endotracheal tubes that favor bacterial proliferation. Other authors [24,25] have shown that SM is ubiquitous and resistant to disinfection and can survive on inanimate surfaces, such as medical devices, and even in antiseptic solutions, for long periods.

Our study highlighted the isolation of SM from medical prewash brush. Although there is no certainty that they are a source of infection, careful epidemiological investigation and environmental surveillance can prevent the transmission of SM to other patients and prevent the onset of dangerous outbreaks. In our case, the brush was carefully washed under running water and disinfected after use. This procedure was probably not sufficient to eradicate any contaminants, or the disinfectant used was not suitable for eliminating SM. We are carrying out some tests to resolve this doubt, in accordance with previous studies [26]. Furthermore, the brush was made from plastic material and previous studies have shown that plastic, being a porous material, can retain both bacteria and SARS-CoV-2, and their persistence on this material lasts for several months [27,28]. Therefore, appropriate disinfection protocols are required.

We believe that the conscious choice of a disinfectant and its use in compliance with the procedures indicated by the manufacturer represent a further intervention to minimize infections related to health care.

Our study has some limitations such as: not having identified the source of infection and the use of PFGE which, despite its high discriminatory power, has long analysis times, often poor inter-gel reproducibility, and requires specific equipment for electrophoresis and computerized image analysis. In our opinion, to control the spread of SM in the hospital setting and to safeguard the health of patients, especially if immunocompromised, targeted preventive measures, such as patient isolation, dedicated hospital staff, and proper hand hygiene are mandatory. Furthermore, environmental microbiological surveillance should always be scheduled, especially during an outbreak, by intensifying the cleaning and sanitizing protocols regarding surfaces and medical equipment [29,30]. In this regard, the conscious choice of a disinfectant and its use in compliance with the procedures indicated by the manufacturer represent a further intervention to minimize infections related to health care [26].

## 5. Conclusions

SM infections currently represent a serious challenge in hospital settings, especially in high-risk wards, such as NICUs and adult ICUs. The identification of clonal relationships by PFGE analysis between clinical and environmental strains allows the rapid implementation of measures to stop the spread of SM. Surveillance programs and proper disinfection procedures are indispensable. Among the preventive interventions, hand hygiene appears to be of particular importance as contaminated hands are a relevant route of transmission. Additionally, the molecular characterization of the strain is essential to detect outbreaks and identify possible sources of infection. The training of health workers on the issues of hand hygiene, use of personal protective equipment, and environmental hygiene are key to reducing the incidence of hospital infections.

## Figures and Tables

**Table 1:** *Serratia marcescens* isolated from clinical and environmental samples in different wards.

SM Strain	Examined Sample	Ward
cs-SM/04	Blood culture	NICU
es-SM/05	Washing brush	NICU
cs-SM/06	CVC	NICU
cs-SM/13	Tracheal aspirate	ICU 2
cs-SM/10	BAL	General surgery
cs-SM/17	Tracheal aspirate	ICU 2
cs-SM/14	Tracheal aspirate	ICU 1
es-SM/18	Faucet handle	ICU 1
es-SM/19	Book support	ICU 1
cs-SM/20	Blood culture	Cardiac surgery
cs-SM/03	Blood culture	NICU
cs-SM/12	BAL	ICU 2
cs-SM/15	Bronchial excreted	ICU 2
es-SM/01	Washbasin	NICU
es-SM/02	Washbasin	NICU
cs-SM/07	Blood culture	Cardiac surgery
cs-SM/08	Blood culture	Internal medicine
cs-SM/09	Blood culture	Cardiac surgery
cs-SM/21	Tracheal aspirate	ICU 1
cs-SM/11	Tracheal aspirate	ICU 1
cs-SM/16	BAL	General surgery
cs-SM/22	Urine culture	ICU 1

BAL, bronchoalveolar lavage; cs-SM: clinical sample-*Serratia marcescens*; CVC, central venous catheter; es-SM: environmental sample-*Serratia marcescens*; /No.: identification number of the studied strains, according to our classification.

**Table 2:** PFGE grouping and % similarity of clinical and environmental strains of *Serratia marcescens* according to sample and ward.

PFGE Group	PFGE Subgroup	SM Strain	Examined Sample	Ward	% Similarity
A	A1	cs-SM/04	Blood culture	NICU	100
A	A1	es-SM/05	Washing brush	NICU	
A	A1	cs-SM/06	CVC	NICU	
A	None	cs-SM/13	Tracheal aspirate	ICU 2	
B	B1	cs-SM/10	BAL	General surgery	95
B	B1	cs-SM/17	Tracheal aspirate	ICU 2	
B	B2	cs-SM/14	Tracheal aspirate	ICU 1	≥96
B	B2	es-SM/18	Faucet handle	ICU 1	
B	B2	es-SM/19	Book support	ICU 1	
B	B2	cs-SM/20	Blood culture	Cardiac surgery	100
B	None	cs-SM/03	Blood culture	NICU	
B	None	cs-SM/12	BAL	ICU 2	
B	None	cs-SM/15	Bronchial excreted	ICU 2	
C	C1	es-SM/01	Washbasin	NICU	97
C	C1	es-SM/02	Washbasin	NICU	
C	None	cs-SM/07	Blood culture	Cardiac surgery	
C	None	cs-SM/08	Blood culture	Internal medicine	
C	None	cs-SM/09	Blood culture	Cardiac surgery	
C	None	cs-SM/21	Tracheal aspirate	ICU 1	
Unique	None	cs-SM/11	Tracheal aspirate	ICU 1	
Unique	None	cs-SM/16	BAL	General surgery	
Unique	None	cs-SM/22	Urine culture	ICU 1	

BAL, bronchoalveolar lavage; cs-SM: clinical sample-*Serratia marcescens*; CVC, central venous catheter; es-SM: environmental sample-*Serratia marcescens*; /No.: Identification No. of *Serratia marcesens* strains; PFGE, pulsed-field gel electrophoresis.

## Data Availability

Not applicable.
